# Author Correction: Limitation of Fermi level shifts by polaron defect states in hematite photoelectrodes

**DOI:** 10.1038/s41467-018-07410-8

**Published:** 2018-11-12

**Authors:** Christian Lohaus, Andreas Klein, Wolfram Jaegermann

**Affiliations:** Surface Science Division, Materials Science Department, TU, Darmstadt, Germany

Correction to: *Nature Communications*; 10.1038/s41467-018-06838-2, published online 17 October 2018

The original version of this Article contained an error in Fig. 2b in which the bottom of the pink-shaded conduction band region was incorrectly positioned at a value of 1.75 eV. The correct conduction band minimum has a value of 2.2 eV. This has now been corrected in both the PDF and HTML versions of the Article.

