# Supplementary material for: Limitation of Fermi level shifts by polaron defect states in hematite photoelectrodes
Source: Nat Commun. 2018 Oct 17;9:4309. doi: 10.1038/s41467-018-06838-2 (PMC6193028; doi:10.1038/s41467-018-06838-2)
Supplement: Supplementary file 1 — Supplementary Information [file 41467_2018_6838_MOESM1_ESM.pdf]

Limitation of Fermi level shifts by polaron defect states in hematite photoelectrodes - Supplementary Information

Lohaus et al.

Supplementary Methods

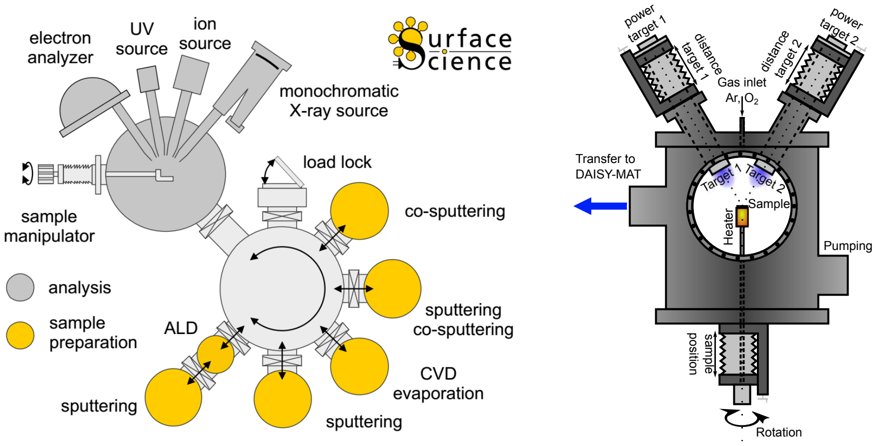

Supplementary Figure 1: Schematic representation of the experimental setup. On the left hand side the DAISY-MAT facility is shown where sample preparation chambers are connected to a Physical Electronics PHI5700 multitechnique surface analysis system for XPS measurements. Sample transfer is possible under UHV conditions. On the right hand side a schematic representation of the co-sputter chamber is shown.

Supplementary Table 1: Deposition conditions for undoped and doped hematite thin films

	Fe_2O_3	$Mg:Fe_2O_3$	$Zr:Fe_2O_3$	$Si:Fe_2O_3$
Target	Fe	Fe/Mg	Fe/Zr	Fe/Si
Power [W]	60	60/15	60/15-45	60/15-30
Sample distance [cm]	8	8/7-14	8/10	8/8-12
Temperature [°C]	RT-600	RT-400	400	
Oxygen content [%]	6-20	8		
Pressure [Pa]	0.5			
Dopant/Fe ratio [%]	-	5.3-26.7	0.4-5.8	2.2-15.5

Supplementary Table 2: Deposition conditions for the contact materials in interface experiments

	ITO	RuO_2	NiO
Target	ITO (10 %Sn)	Ru	Ni
Power [W]	25 (RF)	5 (DC)	40 (DC)
Sample distance [cm]	10	9.4	
Temperature [°C]	RT		
Oxygen content [%]	0	7.5	20
Pressure [Pa]	0.5		

The data acquisition and processing of an interface experiment of hematite and ITO is being represented in Supplementary Figure 2. After a XPS measurement on a bare hematite film ("the substrate") has been performed a thin layer of ITO ("the film") is being deposited on top of the substrate and a XPS measurement follows. As is shown in panel a) and b) this step-wise process is being repeated until no signal from the substrate can be found anymore. Usually, the core-levels of substrate and film are chosen to not overlap in order to acquire independent information from the two materials. From their shift the behavior of the Fermi level in the substrate can be deduced which allows for a determination of band alignment between and band bending in the substrate

and film.[1] In the case of hematite and ITO, however, the Fe2p emission from hematite is overlapped by the $Sn3p_{3/2}$ emission from ITO. The same appears for the oxygen emission as well. This is represented in Supplementary Figure 2 b) by the purple color. For the Fe2p spectrum it is, therefore, necessary to perform a further data processing step in order to remove the $Sn3p_{3/2}$ emission. For this purpose the $Sn3p_{3/2}$ spectrum from the final measurement (VI) has been subtracted from each other spectrum as is being shown in Supplementary Figure 2 c). In order to compensate for the different signal intensity due to the changing film thickness and the different shift of the Fermi level during the course of the experiment the $Sn3p_{3/2}$ spectrum was weighted and shifted according to the development of the $Sn3d_{5/2}$ core-level. The resulting Fe2p spectra are shown in panel d). Their shift of peak position over the course of the experiment can be plotted as is being shown in panel e). The shift does directly correspond to a shift of the Fermi level within the substrate. [1]

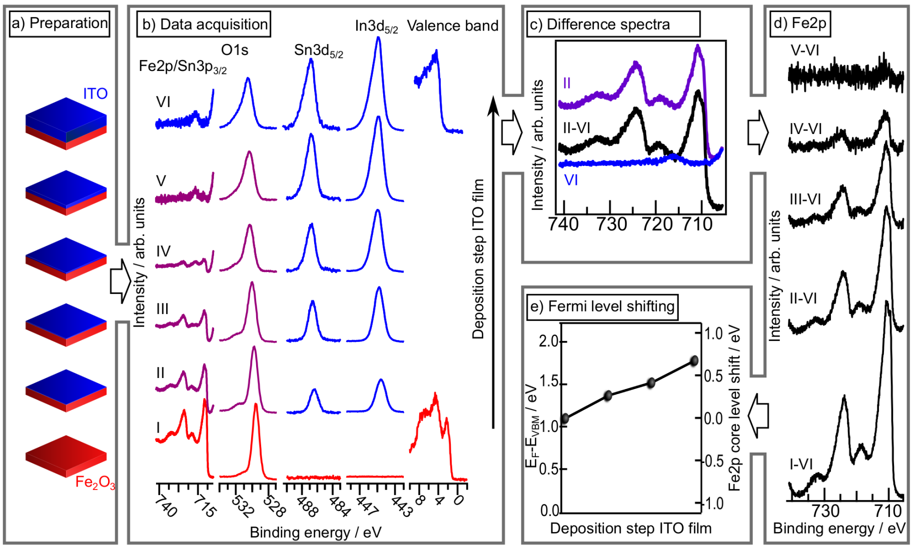

Supplementary Figure 2: Representation of the data acquisition and processing of an interface experiment on the example of hematite and ITO. a) Stepwise ITO deposition on a Fe_2O_3 substrate with XPS measurements in between depositions, b) resulting XP spectra for all relevant core levels (blue: ITO, red: Fe_2O_3 , purple: superposition), c) data processing of the Fe2p peak by substracting the scaled $Sn3p_{3/2}$ spectrum, d) resulting Fe2p spectra, and d) shift of binding the Fe2p binding energy depending on the ITO deposition step corresponding to a shift of the Fermi energy of the Fe_2O_3 substrate.

Supplementary Discussion

The additional XP spectra in Supplementary Figure 3 verify the Fe^{3+} oxidation state for all samples presented here. [2, 3, 4] The shape of the spectra are rather similar, only small differences in the intensity of the "pre-edge" of the $Fe2p_{3/2}$ spectrum (edge towards lower binding energies from main line) for the Mg-doped sample are to be observed. This can be connected to a slightly lower crystallinity within this sample. [2] O1s and valence band spectrum, however, do not show differences to the other samples.

The spectra show different Fermi level positions. This change is accompanied by a shift of the core-level positions as to be expected.

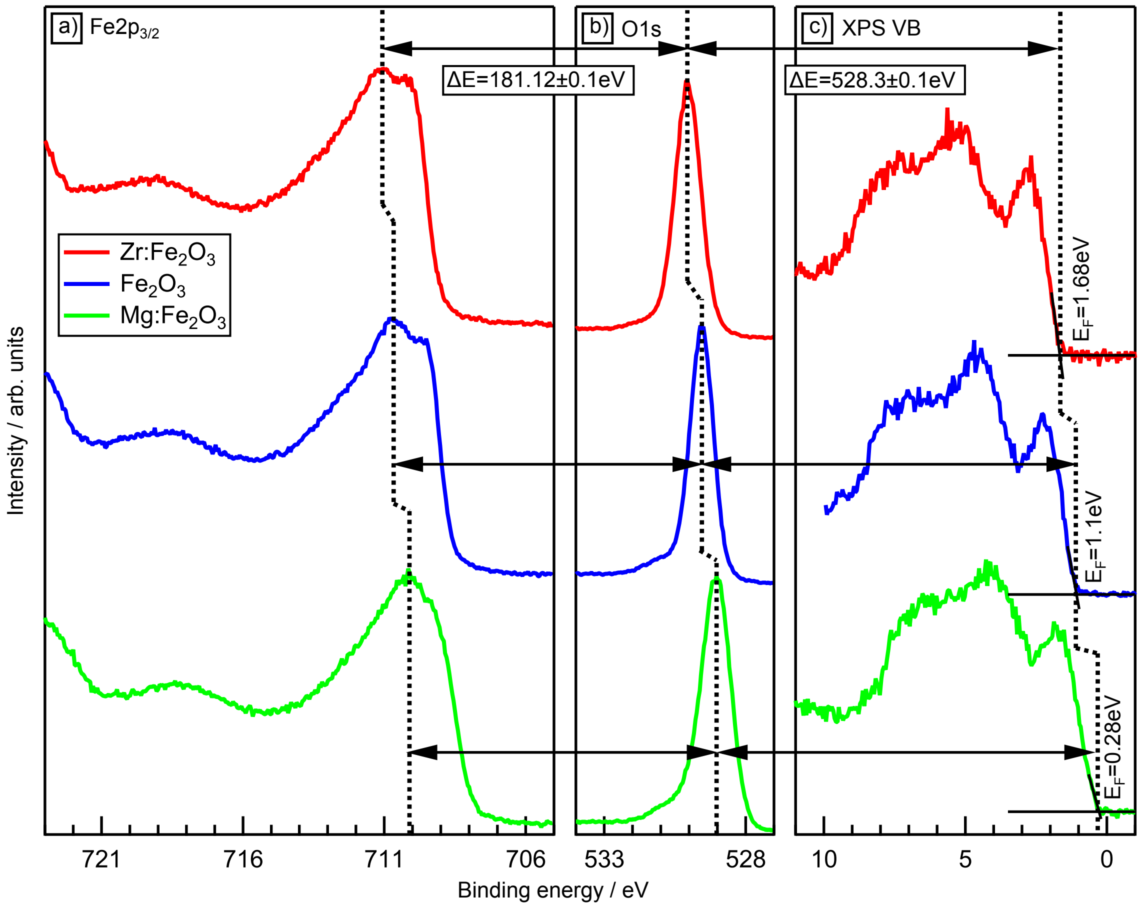

Supplementary Figure 3: X-ray photoelectron emission spectra of hematite. In a)-c) the $Fe2p_{3/2}$-, O1s-core level, and the valence band of three samples with low (Mg-doped), average (undoped), and high (Zr-doped) Fermi level positions.

Interface experiments to the contact materials RuO_2, NiO, and ITO of differently doped films with different contact materials were conducted. The resulting Fermi level positions are displayed in Supplementary Figure 4. Their positions are in excellent agreement with the conclusions of this work. The spectra of the interface experiments included in this work are shown in Supplementary Figure 5 to 7.

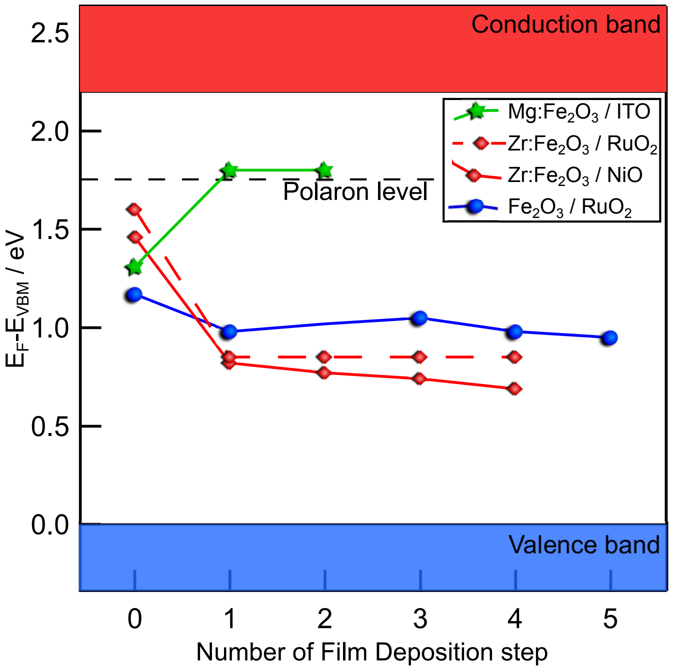

Supplementary Figure 4: Fermi level position of differently doped hematite thin films in interface experiments to different contact materials. The contact material was always grown onto the hematite thin film.

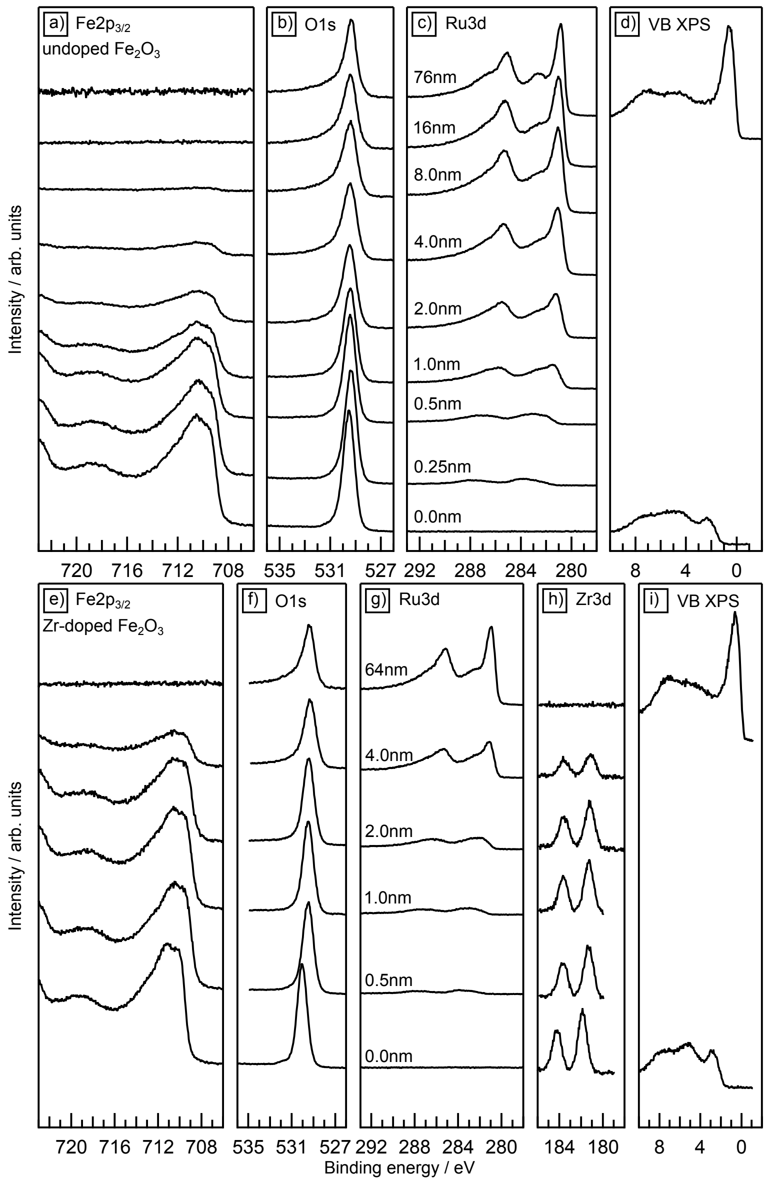

Supplementary Figure 5: XP spectra for interface experiments to RuO_2. Undoped Fe_2O_3 [a)-d)] and Zr-doped Fe_2O_3 [e)-i)] thin films were used a substrate. The calculated thickness of the RuO_2 film assuming a rate of $3\,\mathrm{nm\,min^{-1}}$ is annotated.

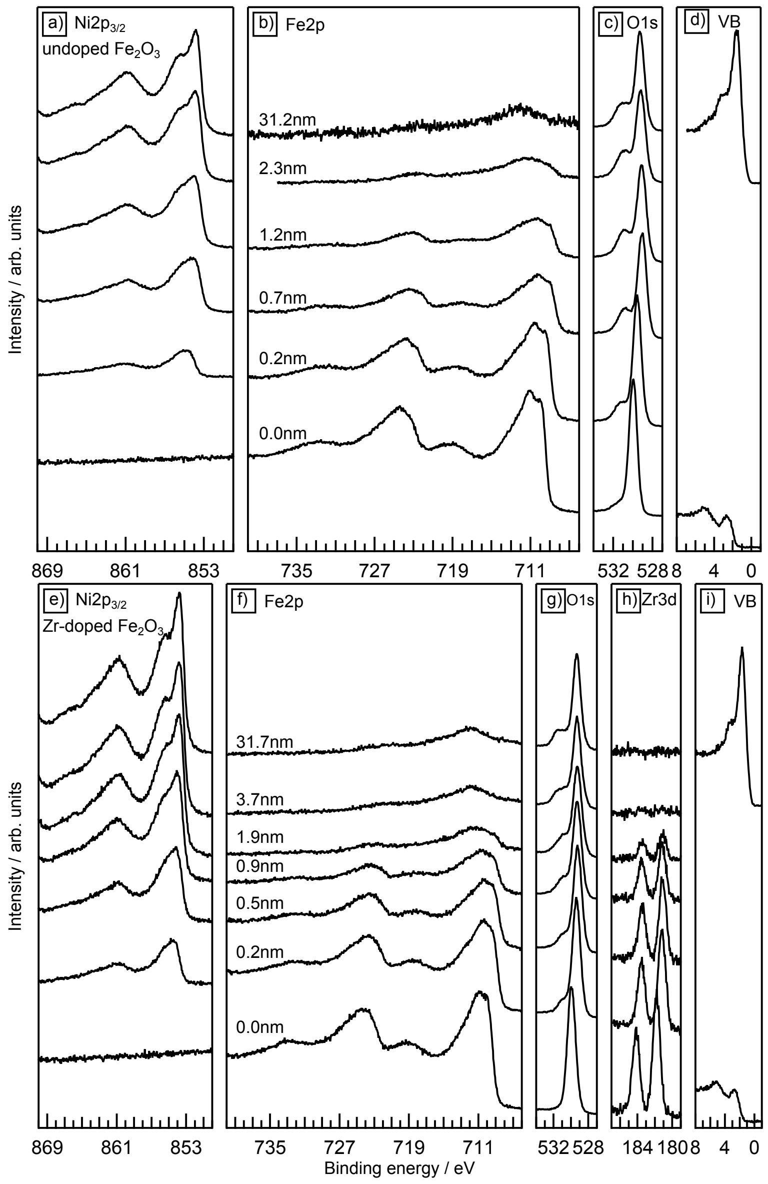

Supplementary Figure 6: XP spectra for interface experiments of to NiO Undoped Fe_2O_3 [a)-d)] and Zr-doped Fe_2O_3 [e)-i)] thin films were used a substrate. The calculated thickness of the NiO film assuming a rate of $1.4\,\mathrm{nm\,min^{-1}}$ is annotated.

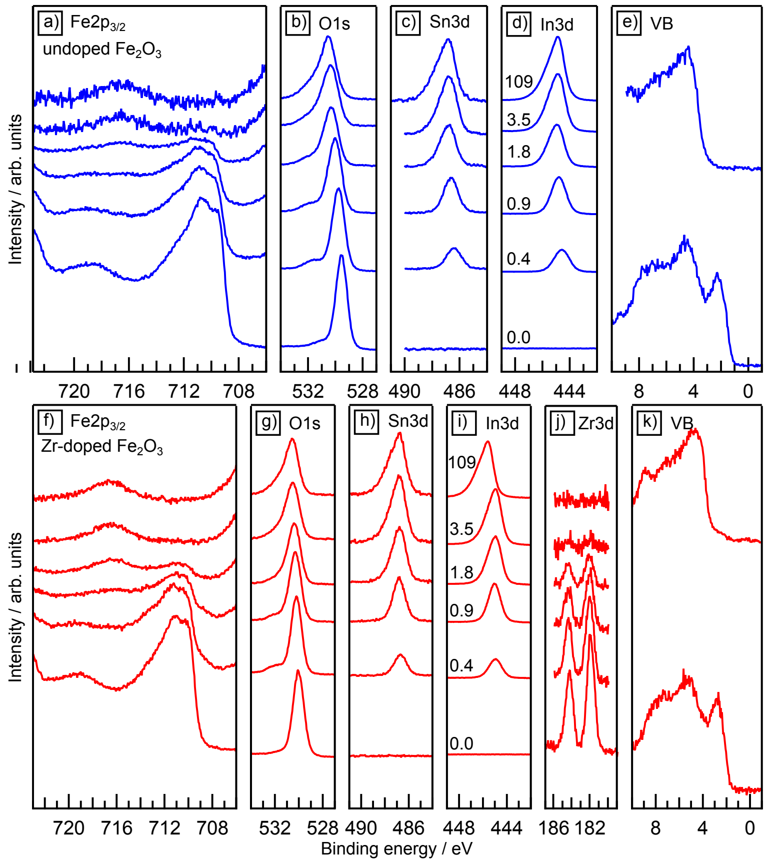

Supplementary Figure 7: XP spectra for interface experiments to ITO .Undoped Fe_2O_3 [a)-e)] and Zr-doped Fe_2O_3 [f)-k)] thin films were used a substrate. The calculated thickness of the ITO film in nanometer assuming a rate of 5.3 nm min^{-1} is annotated.

The interface experiments to $SrTiO_3$ (STO) differed from the experiments to RuO_2, NiO, and ITO as in this single experiment hematite was grown onto a$SrTiO_3$ single crystal. For this purpose, a STO single crystal was heated in 0.5 Pa oxygen at 400 °C for 90 min in order to remove carbon containing surface contamination.

Then, an interface experiment was performed. [1, 5, 6] Hematite was deposited onto STO at 400 °C with 8 % oxygen in the plasma. The accumulated deposition times were 0 s, 5 s, 15 s, 30 s, 60 s, 120 s and 1000 s.
Supplementary Figures 8a)-e) show the evolution of the core-level of hematite and STO starting with the pure STO substrate at the bottom. The position of the Fermi level within STO and hematite was deduced by following the core-level binding energies. The Fermi level positions in dependence on the deposition step are plotted in Supplementary Figure 8f).

The Fe2p core-level spectra in a) are characteristic for Fe_2O_3. There is no strong shift observable and the appearance of the "pre-edge" in the Fe2$p_{3/2}$ main-line suggests that crystalline hematite has been deposited which is expected at the chosen deposition conditions. [2] The same is implied by the upper valence band in e). The final Fermi level position was extracted from a linear extrapolation to the lower region of the edge of the valence band maximum to be at about 1.4 eV.
The core-level from the substrate show a shift of 0.4 eV towards lower binding energies upon the first two depositions of hematite. For further depositions the Fermi level is not shifted anymore.
The positions of the Fermi level in dependence of the deposition step show a Fermi level position in hematite of about 1.5 eV. In STO the Fermi level is initially at about 3 eV and is then shifted to about 2.6 eV. From these values it can be deduced that the valence band of hematite is positioned 1.1 eV above the valence band of STO. A slightly higher value of 1.5 eV was deduced from transitivity considerations. [7, 5]

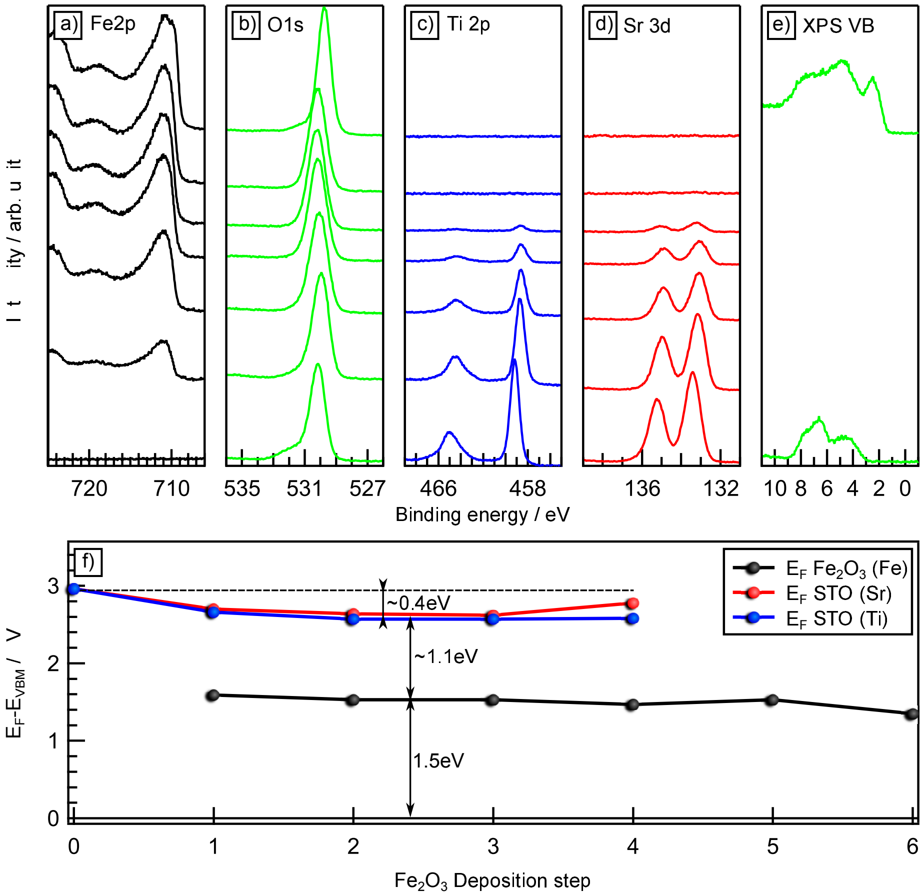

Supplementary Figure 8: Interface experiment of Fe_2O_3 to STO. Hematite was grown in a stepwise process on an STO substrate. Supplementary Figures a)-e) show the growing of the Fe_2O_3 film. In f) the Fermi energy in STO and hematite is plotted in dependency of the deposition step.

The work function dependency on the Fermi level position in differently doped and undoped hematite thin films is presented in Supplementary Figure 9.

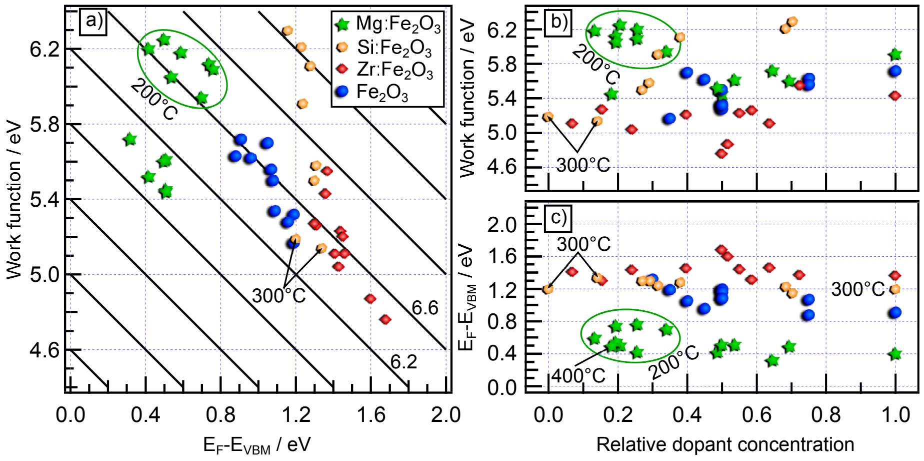

Supplementary Figure 9: Fermi level positions and work functions of differently doped and undoped hematite thin films. In a) the dependency of the work function on the Fermi level position is shown. The diagonal line show constant ionization potentials. Most samples show an ionization potential around 6.6 eV. The deviation of Si- and Mg-doped samples from the range of ionization potential can be attributed to surface segregation due to the high dopant concentration of these samples. The Fermi level position shown here are the same as in Figure 2. The range of Fermi level positions for the different dopants can clearly be distinguished. In b) the dependency of the work function on the relative dopant concentration is presented. c) shows the same Fermi level dependency on the relative dopant concentration. The relative dopant concentration in this regard is the the ratio of the dopant concentration of the respective sample and the maximal dopant concentration of the study on this particular dopant. For Mg, Si, and Zr as dopant the maximal dopant concentration is 8.2 %, 15.5 %, and 5.8 %, respectively. The undoped samples in b) and c) are shown with respect to the maximal oxygen content in the sputter gas during the deposition of 20 %. A variation of the work function and Fermi level position with the composition and deposition conditions can be observed. High oxygen partial pressures tend to lower the Fermi level position and thereby increase the work function.

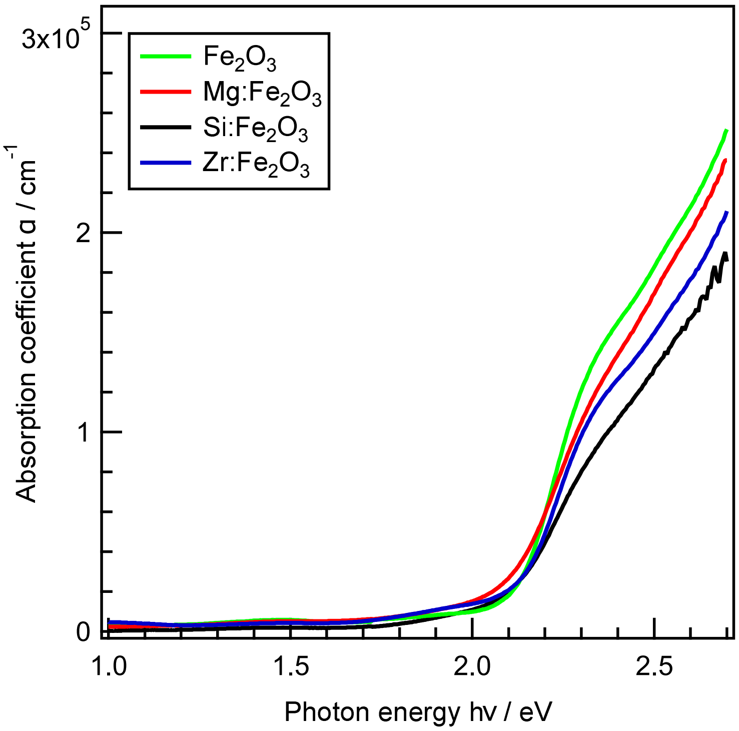

Supplementary Figure 10: Optical absorption coefficient for differently doped and undoped hematite thin films. The absorption coefficient was obtained from transmission and reflection measurements. The optical band gap can be derived from the low energy onset of absorption. It is not changed due to the doping. The optical absorption in the energy region presented here involves Fe3d-states. As a consequence, the smaller absorption coefficient for the doped samples can be assigned to originate from the substitution of iron by the dopant.

Supplementary References

[1] Kraut, E., Grant, R., Waldrop, J. & Kowalczyk, S. Precise determination of the valence-band edge in x-ray photoemission spectra: Application to measurement of semiconductor interface potentials. *Physical Review Letters* **44**, 1620–1623 (1980).

[2] Lohaus, C., Steinert, C., Brötz, J., Klein, A. & Jaegermann, W. Systematic Investigation of the Electronic Structure of Hematite Thin Films. *Advanced Materials Interfaces* **4**, 1700542 (2017).

[3] Fujimori, A., Saeki, M., Kimizuka, N., Taniguchi, M. & Suga, S. Photoemission satellites and electronic structure of Fe_2O_3. *Physical Review B* **34**, 7318–7328 (1986).

[4] Kim, C.-Y., Bedzyk, M. J., Nelson, E. J., Woicik, J. C. & Berman, L. E. Site-specific valence-band photoemission study of $\alpha-Fe_2O_3$. *Physical Review B* **66**, 085115 (2002).

[5] Klein, A. Interface Properties of Dielectric Oxides. *J. Am. Chem. Soc.* **99**, 369–387 (2016).

[6] Schafranek, R., Li, S., Chen, F., Wu, W. & Klein, A. $PbTiO_3/SrTiO_3$ interface: Energy band alignment and its relation to the limits of Fermi level variation. *Physical Review B* **84**, 045317 (2011).

[7] Li, S. *et al.* Intrinsic energy bandalignment of functional oxide. *Phys. Status solidi (RRL)* **8**, 571–576 (2014).
